# Prolonged Time to Brake Following Lower Extremity Injuries

**DOI:** 10.5435/JAAOSGlobal-D-23-00018

**Published:** 2023-04-05

**Authors:** Andrew L. Alejo, Alexander Rascoe, Chang-Yeon Kim, Bryan O. Ren, Matthew T. Hoffa, Isabella M. Heimke, Heather A. Vallier

**Affiliations:** From Northeast Ohio Medical University, Rootstown, OH (Mr. Alejo); School of Medicine, Case Western Reserve University, Cleveland, OH (Dr. Rascoe, Dr. Kim, Dr. Ren, Mr. Hoffa, and Dr. Vallier); and Ohio University College of Medicine, Cleveland, OH (Ms. Heimke).

## Abstract

No guidelines exist for recommending return to driving. This study will examine time to brake (TTB) after lower extremity injuries versus in uninjured people. The potential effect of various types of lower extremity injuries on TTB will be measured.

**Methods:**

Patients with injuries to the pelvis, hip, femur, knee, tibia, ankle, and foot underwent testing using a driving simulator to assess TTB. Comparison was with a control group of uninjured people.

**Results:**

Two-hundred thirty-two patients with lower extremity injuries participated. The majority were in the tibia and ankle regions (47%). Mean TTB for control subjects was 0.74 seconds, compared with 0.83 for injured patients, noting a 0.09-second difference (*P* = 0.017). Left-sided injuries averaged TTB of 0.80 seconds, right-sided injuries averaged TTB of 0.86 seconds, and bilateral injuries averaged TTB of 0.83 seconds, all prolonged versus control subjects. The longest TTB was exhibited after ankle and foot injuries (0.89 seconds) while the shortest was after tibial shaft fractures (0.76 seconds).

**Discussion:**

Any lower extremity injury caused a prolonged TTB compared with control patients. Left, right, and bilateral injuries all had longer TTB. Ankle and foot injuries experienced the longest TTB. Additional investigation is warranted to develop safe guidelines for return to driving.

A recurrent question that arises after a complex lower extremity injury is “when can I drive?” According to the latest US Department of Transportation information, 87% of individuals drive daily commutes by private vehicles and 93% of those individuals drive up to 25 miles each day.^1^ Therefore, the inability to drive a vehicle after lower extremity injury can have an immense socioeconomic effect, possibly precluding return to employment and/or inconveniencing family members and friends to assist with transportation. In fact, a recent study showed an average of 1759.8 lost work hours costing patients more than $64,427 in economic loss because of severe lower extremity injuries.^2^

Often times the patient will seek a response to this question from their surgeon, an answer that is not always easy to discern. Although a review published by the National Highway Traffic Safety Administration has recommendations regarding certain medical procedures and conditions, these are not meant to be used as formal guidelines.^3^ Currently, no standards exist for orthopaedic surgeons to guide return to driving after lower extremity injury. These uncertainties could translate to returning to driving too soon, potentially compromising patient safety, or unnecessarily delaying return to driving, prolonging already detrimental implications. Therefore, establishing a threshold for a safe return to driving and creating guidelines for this would have substantial merit.

Limited prior work has addressed safe time to return to driving after complex lower extremity trauma.^4,5,6,7^ Time to brake (TTB) is the sum of the time necessary to react to a visual stimulus while moving the foot from the accelerator to the brake pedal and applying enough pressure to the brake pedal to stop the vehicle. TTB objectively measures a person's “reaction” time to an object on the road while driving and is considered the current standard for measurement. The purpose of this study was to address the following questions:

1. How does TTB for patients with recent lower extremity injuries compare with TTB for uninjured people?
2. How do various types of lower extremity injuries affect TTB?

## Methods

### Study Population

After obtaining Institutional Review Board approval, 232 patients who had valid driver's licenses and were driving independently before their injury were recruited from a single level 1 trauma center between 2018 and 2021. All had sustained lower extremity injuries to the pelvis ring, acetabulum, proximal femur, femur shaft, knee (patella, tibia plateau), tibia shaft, ankle, and/or foot and were between 18 to 85 years. Patients who were allowed to bear weight on the right leg were approached for inclusion, as long as they did not require assistive braces, splints, or other devices on their right leg. Researchers approached eligible patients in the orthopaedic clinic at their first visit after hospitalization once weight-bearing on the right leg had been allowed. Exclusion criteria included age older than 85 years, no active preinjury driving history, traumatic brain injury, cognitive decline, or inability to speak English. Patients were tested an average of 16 weeks after injury.

Data collected included demographic, injury, and treatment information. A control group of 25 healthy, adult volunteers with no history of previous lower extremity injuries or surgery were tested to establish a normal mean value for TTB.

### Driving Simulation

**Figure 1:**
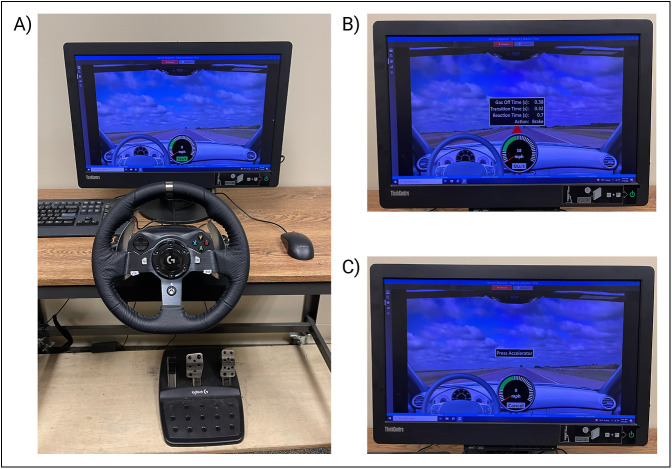
**A**–**C**, Images of the driving simulator setup. The driving simulator setup includes the onscreen driving simulator, wheel, and foot pedals for the brake and accelerator device below (**A**). The onscreen notification after the individual has completed one run of the simulation is shown (**B**); this displays the calculated TTB. The start of the driving test is shown where the patient is instructed to press the accelerator to begin the test (**C**). TTB = time to brake

A computerized driving simulator was assembled using the Vericom Stationary Reaction Timer (Vericom LLC). This system is commonly used as an accelerometer device in driving schools and rehabilitation centers to test driving function. It has also been used in similar studies to assess driving capabilities in a simulated driving experience.^8^ The simulator comprised a digital driving scene displayed on a computer screen, a wheel mounted in front of the computer screen, and accelerator and brake pedals located in the same position as they would be in a car underneath the wheel (Figure 1). On the screen, a speedometer and a timer are shown, both used to calculate the time it takes the patient to halt the vehicle when the brake pedal is pressed.

The patient was taught how the driving simulation would work. They would first have to maintain a speed between 10 and 55 MPH by pressing the accelerator with their foot while their hands were on the wheel. Then, a red triangle would appear on the road at a random time point, and the patient would then have to release the accelerator and move their foot over to the brake pedal to press it down, halting the onscreen vehicle, which would assess their TTB. Once the instructions were explained, the patient could practice the driving simulation up to two times before testing begun to help familiarize them with the simulator. When they felt comfortable to proceed, actual testing began. Each unique patient TTB data set was collected over a series of five runs through the driving simulator. These five runs were averaged to create a mean TTB score. At the end of the five runs, if any outliers (TTB more than twice as large as the average of the trials) were present, the patient would complete an additional run to ensure there were five runs present for the mean calculation. The TTB in this experiment was defined as the time it took for the patient to react to when the stimulus was first presented, to complete pressing of the brake which halted the vehicle.

### Statistical Analysis

The Student *t*-test unpaired with unequal variances and two-tailed (α = 0.05) was used where appropriate with Microsoft Excel version 16.38 (Microsoft). The Student *t*-test unpaired with unequal variances was used to calculate significance of TTB between healthy versus injured patients overall, as well as healthy versus specific injury location of the injured patients (pelvis ring, acetabulum, proximal femur, femur shaft, knee, tibia shaft, ankle, and/or foot). All statistical testing were done two-tailed at an alpha level of 0.05.

## Results

Two hundred thirty-two patients were enrolled to participate. Demographic and injury location data are presented in Table 1. The group of patients consisted of 135 men and 97 women with mean age 46 years. The laterality of the injuries consisted of 97 left-sided injuries, 109 right-sided injuries, and 26 bilateral injuries. Most of the injuries occurred in the tibia and ankle regions (47%), followed by the pelvis/hip (24%), knee/femur (23%), and foot (14%). One hundred ninety-five patients underwent fixation of injuries while 37 had nonsurgical management of orthopaedic injuries. All study patients completed the test uneventfully. None reported new foot or leg pain secondary to the physical aspect of the test.

**Table 1:** Demographics and Injury Features

Total patients n=232	
Average age (SD)	46.0 (16.6)
Sex, n (%)	
Male	135 (58.2)
Female	97 (42.8)
Race, n (%)	
White	159 (68.5)
Black	57 (24.6)
Other/declined	16 (6.9)
Side of injury, n (%)	
Left	97 (41.8)
Right	109 (47.0)
Bilateral	26 (11.2)
Injury location, n (%)	
Pelvis/hip	65 (25.3)
Thigh	23 (8.9)
Knee	32 (12.5)
Lower leg	46 (17.9)
Ankle	75 (29.2)
Foot	16 (6.2)

### Comparison of Injured Patients with Control Subjects

Twenty-five healthy volunteers with no history of lower extremity injury were recruited. These included 17 men and eight women with mean age 38.7 years. Mean TTB for the control group was 0.74 seconds (range, 0.52 to 1.06; SD, 0.16; 95% confidence interval, 0.68 to 0.80). The mean TTB for all injured patients was 0.83 seconds (range, 0.49 to 2.15; SD, 0.25; 95% confidence interval, 0.80 to 0.86). Therefore, a 0.09-second difference between control subjects and injured patients (*P* = 0.017) was noted. Left-sided injuries averaged a TTB of 0.80 seconds, right-sided injuries averaged a TTB of 0.86 seconds, and bilateral injuries averaged a TTB of 0.83 seconds (Table 2). The right-sided and bilateral injuries each resulted in a longer TTB compared with the left-sided injuries.

**Table 2:** Mean TTB is Reported Based on Laterality of Injury

Injury	Mean TTB (s)	*P* Value^a^
Left (n = 97)	0.80	0.15
Right (n = 109)	0.86	0.004
Bilateral (n = 26)	0.83	0.13

TTB = time to brake

a *P* values compare that group with the other injured groups.

### Effects of Various Types of Injuries

Patients with specific injury regions including pelvis/hip, femur/thigh, knee, tibia, ankle, and foot injuries on the right extremity exhibited increased TTB compared with healthy control subjects (range, 0.02 to 0.15 seconds) (Table 3). Of note, the longest TTB (0.93 seconds) was exhibited in ankle (*P* = 0.001) and foot (*P* = 0.002) injuries compared with the shortest TTB (0.78 seconds) exhibited in tibial injuries (*P* = 0.24). Pelvis/hip (*P* = 0.02) and femur/thigh (*P* = 0.01) injuries averaged a TTB of 0.83 seconds.

**Table 3:** Mean TTB is Reported Based on Locations of Injury to the Right Leg Compared With Healthy Control Subjects

Specific injury region	Mean TTB (s)	*P* Value
Pelvis/hip (n = 35)	0.83	0.02
Thigh (n = 13)	0.83	0.01
Knee (n = 17)	0.79	0.29
Lower leg (n = 26)	0.78	0.24
Ankle (n = 5)	0.95	0.001
Foot (n = 9)	0.91	0.002

TTB = time to brake

## Discussion

Safe operation of an automobile requires adequate function of both upper and lower extremities. In 2019, an estimated 151 million emergency department visits occurred in the United States, with over 35 million accounting for injury-related visits.^9^ After complex lower extremity injuries, patients shift their focus on their recovery. Potential criteria to allow a patient to return to driving include weight-bearing capability, conclusion of immobilization, cessation of narcotic use, evaluation by an occupational or physical occupational therapist, or performance on practice drives. Physicians likely rule on the side of caution and do their best to look at their patient holistically before recommending them to return to driving. Currently, no guidelines exist to help physicians assess a patient's true ability to drive safely: therefore, collecting data and validating different metrics would be of substantial merit.

Given the importance of being able to drive, this study was designed to further add supporting data to complex lower extremity injuries and the ability to safely return to driving by using the TTB metric. Although this objective measurement is the current best way to assess driving safely after an injury, it is also a limitation in our study. Objectively measuring a person's ability to drive should also take into account their vision, use of the steering wheel, mental status, and overall performance of driving before their injury, in addition to TTB. None of our study patients had traumatic brain injury or impaired eyesight. The computerized component of the driving simulation was also a limiting factor because it does not completely simulate real driving conditions. Furthermore, these patients were treated at a busy level one trauma center, where they may have felt a time constraint to finish the driving simulation after their outpatient visit with their physician to leave. Finally, we only measured TTB at a single time point after injury. Previous studies^6,10,11^ measured multiple time points after injury and compared each point with one another to show a continuum of recovery for each patient, where eventually, the TTB normalized. TTB on a simulator also does not account for variations inherent in actual operation of a motor vehicle, where some brakes and some driving conditions may require more pressure to affect stoppage of the vehicle.

The purpose of this study was to examine the TTB under a simulated driving experience to see how various complex lower extremity orthopaedic injuries may affect driving ability. Our results demonstrated that TTB is prolonged after lower extremity injuries compared with healthy control subjects. Our control group had no history of lower extremity injury, although they were slightly younger than the patient population (38 vs 46 years). This difference in age is a limitation our study was not able to account for. Our findings in a large patient population are consistent with the expectation that TTB will be increased after an injury, and temporarily limited use of the lower extremities is needed to safely operate a vehicle, consistent with previous studies.

One of these studies conducted by Ho et al^11^ showed that patients with ankle fractures had increased TTB after injury. After 6 weeks of full weight-bearing, 91% of their patients had passed their on-road driving test, which showed that the patient could drive safely. They measured TTB comparably with a driving simulator and using an automatic transmission car in 23 patients. All patients had right ankle injuries that were managed surgically, in which TTB was assessed over various time points postoperatively. We did not obtain serial measurements on our patients; however, we measured patients with ankle fractures within 3 weeks of when they would have begun weight-bearing activity. Similarly, a study led by Jeske et al^12^ showed trends of increased TTB in patients with grade II and III ruptures of lateral ankle ligaments. Four weeks after injury and conservative treatment, patients had a sufficient TTB to be able to drive safely again. They measured TTB similarly with a driving simulator and compared injured patients with control subjects; enrollment of 30 healthy (uninjured) and 30 injured patients was used for this comparison.

In these previous studies, the recommendation to return to driving was as early as 4 weeks and as late as 6 weeks based of their established safe TTB metrics of 0.56, 0.55, and 0.85 seconds.^6,11,12^ Clinically, the reported difference between injured and uninjured TTB measurements being notable is important to note showing a difference in the ability of injured patients being able to halt a vehicle longer than those who were uninjured. This is an important metric that can help further guide physicians into exploring options, such as the driving simulator, to test a patient before they clear them to return to drive. Regarding an appropriate metric for comparison, our uninjured control subjects with a mean TTB of 0.74 seconds demonstrate similar function to the standard range posed by the US Department of Transportation Federal Highway Administration (0.64 to 0.70 seconds). A larger and notable difference was noted between our injured groups and the US Department of Transportation Federal Highway Administration recommendations.^13^

Interestingly, TTB was also prolonged after left-sided injuries. This is an important finding because the left lower extremity is not used in the process of braking a car. This may be explained by hesitation to drive after injury due to general pain or stiffness or the lack of recent driving during recovery time after injury. Driving anxiety after a motor vehicle collision can also be a concern for these groups of patients. Previous work suggests that nearly half of patients who have experienced a motor vehicle collision develop post-traumatic stress disorder.^14^

It is probable that our patients were experiencing various sequelae of their injuries, which may have contributed to long TTB. These include physical stiffness, weakness, deficient proprioception or other sensation of their right and/or left legs, as well as fatigue, anxiety, and other mental health dysfunctions. Possibly, the injured group was slower in TTB before their injury and that subtle physical limitations predisposed some people to incurring an injury event. Regardless of the etiologies of prolonged TTB, it is important to acknowledge the notable limitation in TTB after lower extremity injury and to advise patients accordingly about their impairment.

In addition, the longest TTB occurred after injuries of the ankle and foot. This is not surprising considering these anatomical locations are most intimately involved with braking a car. Injuries to these regions may also be directly affecting the patient's perception of being able to press firmly on the brake pedal when needed because the patient may still view themselves as still recovering or weaker than before the injury. Although TTB for patients with recent pelvis fractures was not as prolonged as the ankle and foot injuries, it is important to note that pelvic injuries can be a large barrier in facilitating a safe return to driving, potentially because of the magnitude of musculoskeletal structures injured and rested during a long recovery time.^15^

## Conclusion

In the largest study to date in this area, prolonged TTB was recorded in patients with complex lower extremity injuries compared with healthy control subjects. Both right-sided and left-sided injuries caused a notable increase in TTB, with right-sided injuries having the greatest effect. Ankle, pelvis, hip, and thigh injuries were associated with the longest TTB. Our research suggests that additional work in this area, including TTB measurements, may be needed to aid physicians in assessing their patients for a safe and expeditious return to driving.

## References

[1] Administration USDoTFH: NextGen NHTS national OD data, 2020. https://nhts.ornl.gov/od/. Accessed September 1, 2022.

[2] Levy JF Reider L Scharfstein DO : The 1-year economic impact of work productivity loss following severe lower extremity trauma. J Bone Joint Surg 2022;104:586-593. 10.2106/JBJS.21.00632 35089905

[3] Dobbs BM Wodzin E Vegega M: Medical Conditions and Driving: A Review of the Literature, 2005. https://rosap.ntl.bts.gov/view/dot/1902. Accessed September 1, 2022.

[4] Egol KA Sheikhazadeh A Koval KJ: Braking function after complex lower extremity trauma. J Trauma 2008;65:1435-1438. 19077638 10.1097/TA.0b013e31811eaab8

[5] Egol KA Sheikhazadeh A Mogatederi S Barnett A Koval KJ: Lower-extremity function for driving an automobile after operative treatment of ankle fracture. J Bone Joint Surg Am 2003;85:1185-1189. 12851340 10.2106/00004623-200307000-00001

[6] McDonald EL Shakked R Nicholson K : Return to driving after foot and ankle surgery: A novel survey to predict passing brake reaction time. Foot Ankle Spec 2021;14:32-38. 31904291 10.1177/1938640019890970

[7] von Bernstorff M Bausenhart F Rapp J Feierabend M Ipach I Hofmann UK: Evaluation of braking performances of patients with osteoarthritis of the knee or hip: Are there alternatives to a brake simulator? Acta Orthop Traumatol Turc 2021;55:42-47. 33650510 10.5152/j.aott.2021.19041 PMC7932726

[8] Kim CY Wiznia DH Averbukh L : PROMIS computer adaptive tests compared with time to brake in patients with complex lower extremity trauma. J Orthop Trauma 2016;30:592-596. 27380397 10.1097/BOT.0000000000000645

[9] Cairnes CK Santo L: National hospital ambulatory medical care survey: 2018 emergency department summary tables, 2018. https://www.cdc.gov/nchs/data/nhamcs/web_tables/2018-ed-web-tables-508.pdf. Accessed September 1, 2022.

[10] Burnham M Wright A Kane TJK Kikuchi CK: When do patients return to driving after outpatient foot and ankle surgery? Hawaii J Health Soc Welf 2022;81:13-15. PMC8941613 35340939

[11] Ho SWL Yam M Chan ML Kwek EBK: Return to car driving is safe 6 weeks after operative treatment of right ankle fractures. Arch Orthop Trauma Surg 2018;138:1691-1697. 30229342 10.1007/s00402-018-3037-3

[12] Jeske HC Hirnsperger C Perwanger F : Break reaction time after conservatively treated ligament ruptures of the ankle. Injury 2021;52:2463-2468. 33762092 10.1016/j.injury.2021.02.061

[13] Richard CCJL Brown JL: Task Analysis of Intersection Driving Scenarios: Information Processing Bottlenecks, 2005. https://www.fhwa.dot.gov/publications/research/safety/06033/06033.pdf. Accessed September 1, 2022.

[14] Fekadu W Mekonen T Belete H Belete A Yohannes K: Incidence of post-traumatic stress disorder after road traffic accident. Front Psychiatry 2019;10:519. 31379631 10.3389/fpsyt.2019.00519 PMC6659351

[15] Tullington JE Blecker N: Pelvic Trauma. Treasure Island, FL: StatPearls, 2022. 32310530

